# Litigation in Anesthesia and Intensive Care Units: An Italian Retrospective Study

**DOI:** 10.3390/healthcare9081012

**Published:** 2021-08-07

**Authors:** Emiliano Petrucci, Alessandro Vittori, Marco Cascella, Alessandro Vergallo, Gilberto Fiore, Antonio Luciani, Barbara Pizzi, Giulia Degan, Vittorio Fineschi, Franco Marinangeli

**Affiliations:** 1 Department of Anesthesia and Intensive Care Unit, San Salvatore Academic Hospital of L’Aquila, 67100 L’Aquila, Italy; petrucciemiliano@gmail.com; 2 Department of Anesthesia and Critical Care, ARCO ROMA, Ospedale Pediatrico Bambino Gesù IRCCS, Piazza S. Onofrio 4, 00165 Rome, Italy; 3 Department of Anesthesia and Critical Care, Istituto Nazionale Tumori—IRCCS, Fondazione Pascale, 80131 Naples, Italy; m.cascella@istitutotumori.na.it; 4 Department of Anesthesia and Intensive Care, Spedali Civili di Brescia, 25123 Brescia, Italy; vergallo@aaroiemac.it; 5 Department of Anesthesia and Intensive Care, Hospital of Santa Croce di Moncalieri,10024 Turin, Italy; gilberto.fiore@gmail.com; 6 Department of Anesthesia and Intensive Care Unit, SS Annunziata Hospital of Sulmona (L’Aquila), 67039 L’Aquila, Italy; luciani.ant@gmail.com (A.L.); degan.giulia@gmail.com (G.D.); 7 Department of Anesthesia and Intensive Care Unit, SS Filippo and Nicola Academic Hospital of Avezzano (L’Aquila), 67051 L’Aquila, Italy; bpizzi@hotmail.it; 8 Department of Anatomical, Histological, Forensic Medicine and Orthopedic Sciences, Sapienza University of Rome, 00185 Rome, Italy; vittorio.fineschi@uniroma1.it; 9 Department of Anesthesiology, Intensive Care and Pain Treatment, University of L’Aquila, 67100 L’Aquila, Italy; francomarinangeli@gmail.com

**Keywords:** anesthesia, litigation, claim, intensive care, malpractice, hospital

## Abstract

Anesthesiologists consider professional insurance and its medico-legal problems as a remarkable aspect of their job. “Associazione Anestesisti Rianimatori Ospedalieri Italiani—Emergenza ed Area Critica” (AAROI-EMAC) is the Italian professional association of anesthesiologists and intensivists that works to train its subscribers on safety measures. This is a retrospective observational study on an insurance complaints database for anesthetic accidents that result in injuries to patients. The analyzed period runs from 1 January 2014 to 31 December 2016. A total of 1309 complaints related to 873 insurance claims were analyzed. Criminal complaints comprised 805 (64.4%) of the total, and civil complaints were 445 (35.6%). The iatrogenic damage claimed included: death (58% of the cases); peripheral nerve damage (8%); spinal cord injuries (5%); unspecified injuries (7%); dental damage (4%); infections (3%); needing second surgical procedure (2%); and other injuries (13%). There is a statistical significance between the size of the hospital and the number of the claims: small hospital complaints comprised 40.1% of the cases, while complaints against medium-sized and large hospitals constituted 20.6% of the cases (χ^2^GL = 8 = 39.87, *p* = 0.00). In Italy, anesthesiologists and intensivists are often involved in litigation even when they are not directly responsible for iatrogenic injuries, and the most frequent claims in ICU are related post-operative complications.

## 1. Introduction

Modern surgical departments are characterized by a high degree of automation that can improve the quality of care, simplify clinical workflows, and mitigate equipment-related incidents and human errors [1]. The operating room (OR) is a particularly high-risk environment, prone to surgical and anesthetic accidents with resulting patient injuries. Anesthesiologists consider professional insurance and medico-legal problems as particularly sensitive topics as they develop their specialty [2].

Medical malpractice is a growing field for forensic pathologists, and forensic autopsies are a mandatory step in the judicial evaluation of suspected medical malpractice. Reliable national and international registers about medical malpractice are still missing, and, nowadays, the necroscopic archives are one of the best sources of data about this complex phenomenon.

Mortality risk associated with anesthesia has been the subject of extensive research for many decades [3,4,5,6,7,8,9,10,11,12,13].

The deaths directly attributable to anesthesia are 64 for every 100,000 procedures (the most common causes are due to the anesthetic agents, the intrinsic characteristics of the patients, and the types of providers) [14].

The report by Beecher and Todd helped identify anesthesia safety as a public health problem [14]. The study calls attention to anesthesia mortality risk, as underlined in studies conducted in Europe, Japan, and Brazil [3,15,16,17].

In Italy, there is a lack of data and statistics regarding anesthesia mortality, which is compounded by several factors, though improvements in anesthesia safety have made anesthesia-related deaths rare events. Studying rare events usually requires large sample sizes and considerable resources. Furthermore, there is no established national surveillance data system for monitoring anesthesia mortality. The national data available are related to less comprehensive studies and reports from the National Agency of Insurance Companies, which leaves the data incomplete and not very specific [18,19,20].

The Italian Associazione Anestesisti Rianimatori Ospedalieri Italiani—Emergenza ed Area Critica (AAROI-EMAC) is the Italian syndicate and organization of anesthesiologists and intensive care unit physicians. AAROI-EMAC subscribers are entitled to benefits from the insurance broker that are provided by the syndicate.

The aim of this study is to analyze the database of insurance claims in order to have a first, albeit partial, line of evidence about the Italian epidemiology of the related perioperative medico-legal problems and the surgery/anesthesia-related morbidity.

## 2. Materials and Methods

This is a retrospective observational study on insurance complaints database for anesthetic accidents that result in injuries to the patients. The analyzed period runs from 1 January 2014 to 31 December 2016.

This research was approved by local health authorities, in accordance with the STROBE Statement for observational studies: protocol number 57283/15 Ethics Committee of L’Aquila and Teramo.

In Italy, there are 21,001 anesthesiologists and intensive care unit physicians; 10,818 are AAROI-EMAC’s (Associazione Anestesisti Rianimatori Ospedalieri Italiani—Emergenza ed Area Critica subscribers) [20].

Written informed consent and permission for personal data analysis and potential publication were obtained by the insurance subscribers, at the time of contract stipulation.

The following data were analyzed: demographic characteristics of the subscribers; report type (criminal or civil); modality of complaint involvement; iatrogenic injury; type of damage (related to anesthetic procedures, intensive care unit procedures, or surgical procedures); in-hospital length of stay, private or non-private practices; geographical distribution of the hospitals (North: Piedmont, Aosta Valley, Liguria, Lombardy, Trentino Alto Adige, Veneto, Friuli-Venezia Giulia and Emilia Romagna; Central: Tuscany, Umbria, Marche and Lazio; South: Abruzzo, Molise, Campania, Puglia, Basilicata, Calabria, Sicily and Sardinia); and distribution of complaints per bed (in accordance with the database of the Italian Ministero della Salute) [19].

The data were analyzed on the basis of geographical location because, in Italy, the regions have large decision-making power regarding the organization of the health system in their area of competence [21]. Furthermore, the socio-economic characteristics between the various Italian regions are sometimes very dissimilar [22].

Reports that were completed in an incomprehensible way were duplicated, and those that contained inconsistent information (for example date errors) were excluded from the analysis.

This manuscript adheres to the applicable STROBE guidelines.

### Statistical Analysis

The data were reported and analyzed anonymously. The primary outcome was defined and established a priori at initiation of the study design and accompanying statistical analysis and whether subgroup or sensitivity analyses were identified and established a priori. All the statistical evaluations were performed using the STATA 14 software package.

The statistical association between variables was analyzed by χ2 test assuming a level of statistical significance <0.05.

## 3. Results

**Figure 1:**
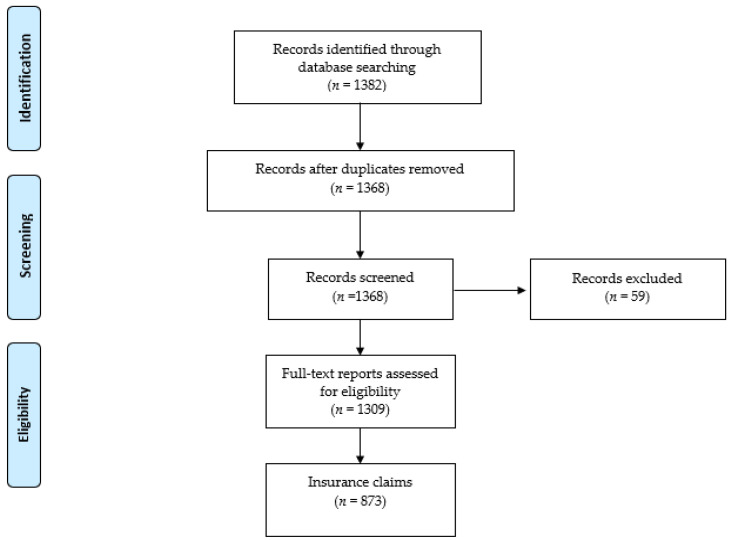
Flow chart of the study.

A total of 1382 complaints were recorded, from 1 January 2014 to 31 December 2016. Of these complaints, 73 were excluded because 1 did not meet the inclusion criteria, 55 were not clearly reported data, 14 were duplicated, and 4 were inconsistent (Figure 1). The remaining 1309 data reports, related to 873 insurance claims, were analyzed.

A decreasing number of claims were noted from 1 January 2014 (540 insurance claims) to 31 December 2016 (384 insurance claims). A total of 795 complaints (61%) involved male anesthesiologist and intensive care unit physicians, while 514 claims (39%) involved female specialists.

The insurance claims against private practice physicians comprised 9.5% of the analyzed reports, while 76.2% were related to specialists in public practice.

The modality of complaint involvements was team responsibility in 52.62% of the cases (652); direct responsibility in 20.01% (249); performing the anesthetic evaluation in 0.97% (12); for intervention as a second anesthesiologist in 0.56% (7); and, for other reasons, 1.37% (17).

The criminal complaints numbered 805 (64.4%), with civil complaints at 445 (35.6%).

The disputed damage in the majority of the cases was death in 962 cases (74.5%); in 313 cases (24.2%), there were complaints about serious injuries; and in 17 cases (1.31%), there were complaints about other types of injuries (10 cases of moral damage, 6 cases of damage to objects, and 1 case of false ideology).

A total of 268 (34%) insurance claims were related to general anesthesia execution; 137 (17.3%) claims were related to the intensive care unit stay, 103 (13%) to spinal or epidural anesthesia, 94 (11.9%) to out-of-hospital rescues, 91 (11.5%) to peripheral nerve blocks, 75 (9.5%) to counseling procedures, and 22 (2.8%) to pain therapy.

### 3.1. Analysis of Demographic Characteristics of the Anesthesiologists Cited in Complaints

The anesthesiologists involved in complaints were 50 years old (SD ± 10.6) on average (60% male and 40% female). They had fewer than 5 years of work experience in 22.4% of the cases; in 37.4% of the cases, they worked from 5 to 20 years, and in 40% of the cases, they had more than 20 years of work experience.

In 77.6% of the cases, they had a no private practice, while in 22.4% of the cases, they had a private practice.

### 3.2. Analysis of Geographic Distribution of the Adverse Events

A total of 1309 complaints were analyzed: 388 were for hospitals in Northern Italy, 297 in Central Italy, and 624 in the Southern part of the country; 637 claims referred to a medium hospital, 420 to a large hospital, and 252 to a small one.

**Figure 2:**
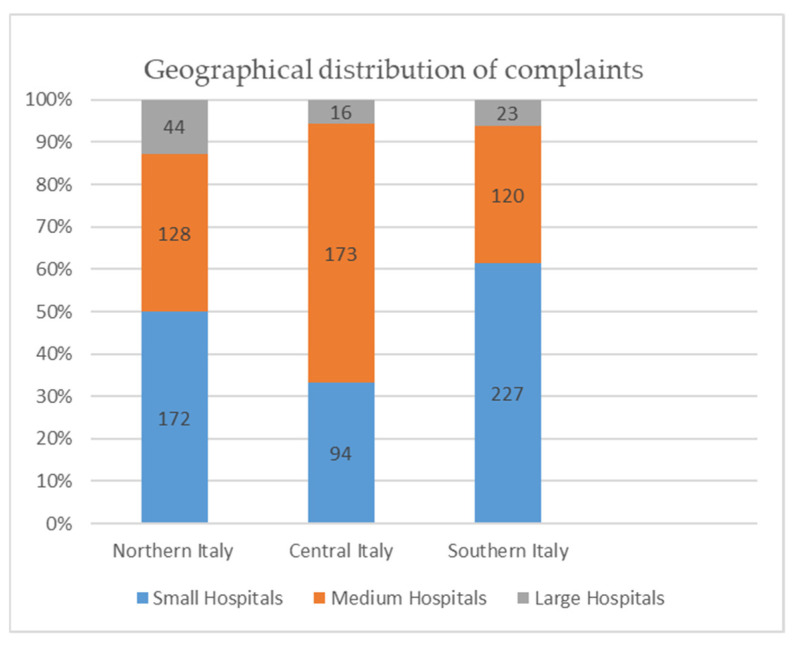
Geographical distribution of complaints.

The geographical distribution of complaints per bed were: 172 for small hospitals (less than 120 beds), 128 for medium hospitals (from 120 to 550 beds), and 44 for large hospitals (more 550 beds) in Northern Italy; 94 for small hospitals, 173 for medium hospitals, and 16 for large hospitals in Central Italy; 227 for small hospitals, 120 for medium hospitals, and 23 for large hospitals in Southern Italy (Figure 2). These complaints were analyzed according to the type of hospital where the individual specialists work, combining those complaints that involved several specialists.

There was a statistical significance between the size of the hospital and the number of the claims; small hospitals received 40.1% of the claims, while medium and large hospitals were the sources of complaints in 59.9% of the cases (χ^2^GL = 8 = 39.87, *p* = 0.00).

### 3.3. Analysis of Insurance Claims Related to Anesthetic Procedures

**Figure 3:**
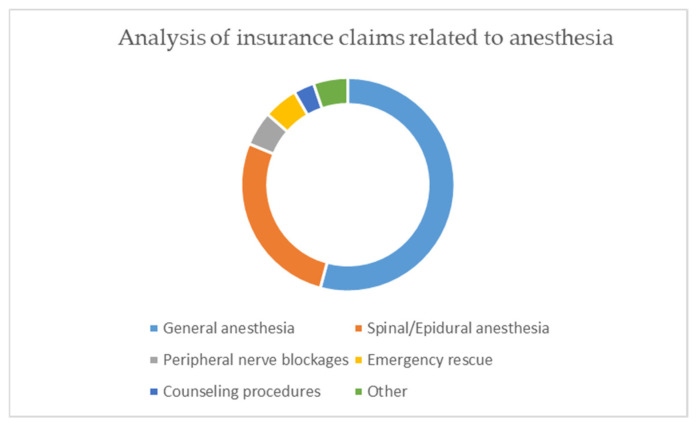
Analysis of insurance claims related to anesthesia.

A total of 258 insurance claims were recorded; they related, in 52% of the cases, to general anesthesia procedures; in 26%, to spinal or epidural anesthesia; in 5%, to peripheral nerve blocks; in 5%, to emergency rescue; in 3%, to counseling procedures; and in 5%, to other reasons (Figure 3).

The complaints related to general anesthesia adverse events referred to the moment of induction (100 cases, 38.1%), to the general anesthesia maintenance period (100 cases 38.1%), to awakening procedures (32 cases, 14.4%), and to post-anesthesia (19 cases, 7.4%) and pre-anesthesia care (7 cases, 2%).

The iatrogenic damage claimed was death (58% of the cases), peripheral nerve damage (8%), spinal cord injuries (5%), unspecified injuries (7%), dental damage (4%), infections (3%), necessity of a second surgical procedure (2%), and other injuries (13%).

**Figure 4:**
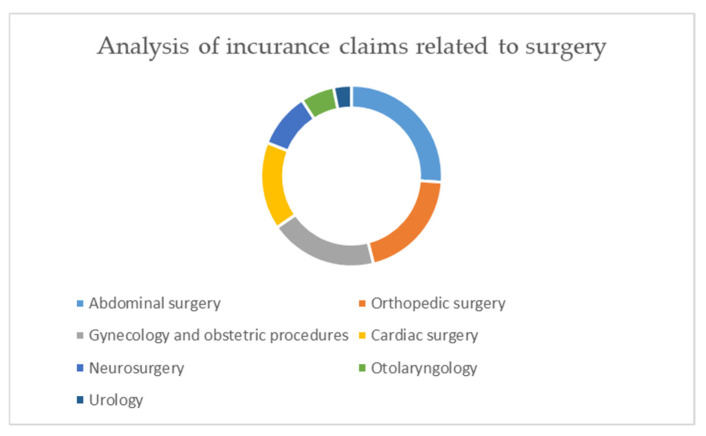
Analysis of insurance claims related to surgery.

The iatrogenic injuries were related to: abdominal surgery (99 cases, 26.1%), orthopedic surgery (76 cases, 20%), gynecology and obstetric procedures (73 cases, 19.3%), cardiac surgery (59 cases, 15.6%), neurosurgery (37 cases, 9.8%), otolaryngology (23 cases, 6%), and urology (12 cases, 3.2%) (Figure 4).

A total of 138 adverse events were reported during the post-operative period: 118 cases (85%) during a post-operative stay in the intensive care unit and 20 cases during a stay in the ward (15%).

### 3.4. Analysis of Insurance Claims Related to a Stay in the Intensive Care Unit

**Figure 5:**
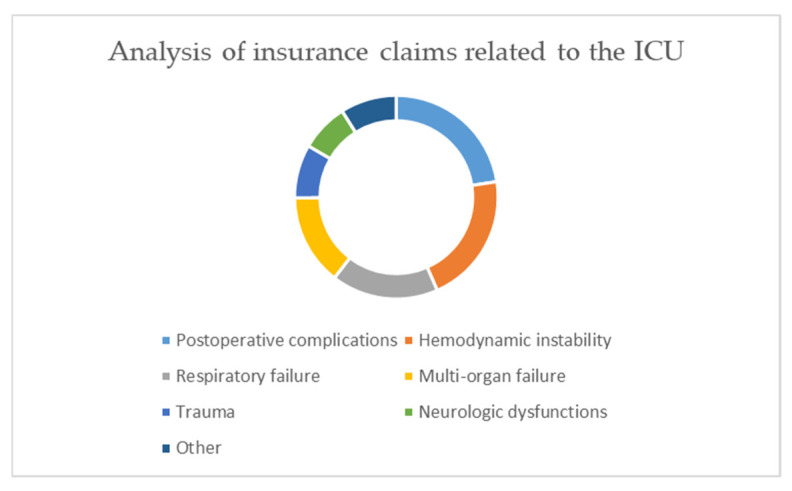
Analysis of insurance claims related to the Intensive Care Units.

Litigation concerning patients in the ICU (Intensive Care Unit) (235 cases) was related to: postoperative complications (53 cases, 22.3%), hemodynamic instability (49 cases, 20.9%), respiratory failure (40 cases, 17%), multi-organic failure (34 cases, 14.5%), trauma (20 cases, 8.5%), neurologic dysfunctions (18 cases, 7.7%), and other reasons (21 cases, 8.9%) (Figure 5).

## 4. Discussion

This report shows that, from 1 January 2014 to 31 December 2016, a decreasing number of claims were filed. Our data supported the hypothesis that inexperience of the anesthesiologist is related to a higher probability of claims for iatrogenic patient injuries, especially in some areas of super-specialization [23,24].

On the other hand, lengthy work experience could be related to reduced attention by the anesthesiologist during the perioperative period, resulting in a lower-quality decision-making process [25,26,27].

In Southern Italy, the number of claims shows an increasing trend inversely proportional to the size of the hospital; a similar but less pronounced trend can also be appreciated in the Central and Northern sections of the country. However, it is necessary to point out that, in Southern Italy, there are more small hospitals than in the other regions of the country.

Based on our report, anesthesiologists are involved in complaints even when the litigation is related to surgical procedures, especially for postoperative complications [25,26,27,28]. The iatrogenic injuries cited in the majority of complaints were related to abdominal surgery, in accordance with the data reported by Genovese et al. and Casali et al. A considerable number of claims were reported for orthopedic surgery, as well as gynecological and obstetric procedures [29,30].

Defensive medicine is a normal practice among the Italian physicians, according to international literature suggestions [28,31]. In fact, in 77.7% of the cases, physicians prescribed unnecessary laboratory examinations; in 72.8%, they registered unnecessary medical records; and in 67.3%, they requested unnecessary specialist consultancy, as reported in a survey by the Italian Society of Surgeons [32].

It is possible to argue that this practice of defensive medicine by physicians is related to the rising instances of litigation between patients and the Italian National Health System. Patients perceive the physicians, especially the anesthesiologists, as representatives of the Health System, so they direct their frustration against the system on the physician.

Another reason for the increase in defensive medicine is the Italian law. Unlike other countries, physicians in Italy are liable for both civil and criminal prosecution. In addition, when there is a criminal conviction, the offended party can use the sentence to initiate civil proceedings. This is burdened by the fact that Italy, among very few countries in the world, provides for the possibility of a criminal measure even when the criminal act was not voluntary but only negligent. Thus, insurance protection is limited to the economic aspects of litigation, not to the judicial freedom of the doctors. To this must be added the fact that civil and criminal proceedings take a long time, which require a heavy economic and emotional commitment [33].

Alarmist media coverage of medical malpractice cases contributes to the creation of a distorted idea of the risk associated with medical procedures, especially in emergency situations, resulting in an increase in claims and distrust of the health system and physicians.

In addition, web resources showcase news of medical science progress that often cannot be realized in all health centers, especially those with fewer beds.

A limit of this report is the timeframe of the research, which involves only three years of insurance claim reports. We also neglected to analyze data from other insurance brokers and the outcomes of litigation, but this decision was made with careful consideration. AAROI-EMAC (Associazione Anestesisti Rianimatori Ospedalieri Italiani—Emergenza ed Area Critica) created a national network of training and simulation centers to improve the anesthesiologists’ decision-making process and multidisciplinary approach, in case of adverse events during elective medical procedures and in emergency settings.

The data analyzed here came from a homogenous database: in this way the added value of this paper is testing the results of courses organized by AAROI-EMAC.

It would be desirable to create one database for all insurance brokers to obtain clear and complete data about claims and litigation outcomes for a complete picture. This could be useful to improve the work and attitude of anesthesiologists and intensive care unit physicians.

## 5. Limitations

Our study shows several limitations. The data in our possession, even if homogeneous and of quality, come only from the AAROI_EMAC (Associazione Anestesisti Rianimatori Ospedalieri Italiani—Emergenza ed Area Critica) database. The data of the Italian anesthesiologists/intensivists who are not registered with the AAROI-EMAC escape our observation. Unfortunately, such data are currently not available because there is no national database. In the study, we refer only to civil and criminal complaints but we do not have complete data regarding the damages awarded and the sentences imposed. In fact, the time period of three years is not sufficient to be able to relate complaints to compensation and sentences. It would therefore be desirable to plan of a long-term prospective study to correlate these data. Another limitation is that of not having, for each anesthesiologist registered with the AAROI, a case history updated by the annual activity. Unfortunately, in Italy, not all health facilities have a digital collection of these data.

## 6. Conclusions

This report is the first step toward the creation of an Italian national insurance claims database for anesthesiologists and intensive care unit physicians, which could improve training to obtain the best management practices by physicians when adverse events occur and in emergency settings. On the basis of our data, although partial, some points of interest emerge: the involvement of anesthesiologists as well as surgeons for surgical complications, the high number of claims in small hospitals in Southern Italy, and the fact that the most frequent claims in ICU (Intensive Care Unit) are related to post-operative complications.

Finally, taking into account the limitations of this study, further studies are needed to support our conclusions.

## Data Availability

The data are available from AAROI-EMAC and may be requested within reason.

## References

[1] Kasparick M., Schmitz M., Andersen B., Rockstroh M., Franke S., Schlichting S., Golatowski F., Timmermann D. (2018). OR.NET: A Service-Oriented Architecture for Safe and Dynamic Medical Device Interoperability. Biomed. Tech..

[2] Dercq J.P., Smets D., Somer A., Desantoine D. (1998). A Survey of Belgian Anesthesiologists. Acta Anaesthesiol. Belg..

[3] Li G., Warner M., Lang B.H., Huang L., Sun L.S. (2009). Epidemiology of Anesthesia-Related Mortality in the United States, 1999–2005. Anesthesiology.

[4] # Closed Claims Project Shows Safety Evolution. https://www.apsf.org/article/closed-claims-project-shows-safety-evolution/.

[5] Arbous M.S., Grobbee D.E., van Kleef J.W., de Lange J.J., Spoormans H.H., Touw P., Werner F.M., Meursing A.E. (2001). Mortality Associated with Anaesthesia: A Qualitative Analysis to Identify Risk Factors. Anaesthesia.

[6] Hove L.D., Steinmetz J., Christoffersen J.K., Møller A., Nielsen J., Schmidt H. (2007). Analysis of Deaths Related to Anesthesia in the Period 1996-2004 from Closed Claims Registered by the Danish Patient Insurance Association. Anesthesiology.

[7] Cranshaw J., Gupta K.J., Cook T.M. (2009). Litigation Related to Drug Errors in Anaesthesia: An Analysis of Claims against the NHS in England 1995–2007. Anaesthesia.

[8] Auroy Y., Narchi P., Messiah A., Litt L., Rouvier B., Samii K. (1997). Serious Complications Related to Regional Anesthesia: Results of a Prospective Survey in France. Anesthesiology.

[9] Michel P., Quenon J.L., de Sarasqueta A.M., Scemama O. (2004). Comparison of Three Methods for Estimating Rates of Adverse Events and Rates of Preventable Adverse Events in Acute Care Hospitals. BMJ.

[10] Zegers M., de Bruijne M.C., Wagner C., Hoonhout L.H.F., Waaijman R., Smits M., Hout F.a.G., Zwaan L., Christiaans-Dingelhoff I., Timmermans D.R.M. (2009). Adverse Events and Potentially Preventable Deaths in Dutch Hospitals: Results of a Retrospective Patient Record Review Study. Qual. Saf. Health Care.

[11] de Vries E.N., Ramrattan M.A., Smorenburg S.M., Gouma D.J., Boermeester M.A. (2008). The Incidence and Nature of In-Hospital Adverse Events: A Systematic Review. Qual. Saf. Health Care.

[12] Tartaglia R., Albolino S., Bellandi T., Bianchini E., Biggeri A., Fabbro G., Bevilacqua L., Dell’erba A., Privitera G., Sommella L. (2012). Adverse events and preventable consequences: Retrospective study in five large Italian hospitals. Epidemiol. Prev..

[13] Lienhart A., Auroy Y., Péquignot F., Benhamou D., Warszawski J., Bovet M., Jougla E. (2006). Survey of Anesthesia-Related Mortality in France. Anesthesiology.

[14] Beecher H.K., Todd D.P. (1954). A Study of the Deaths Associated with Anesthesia and Surgery: Based on a Study of 599, 548 Anesthesias in Ten Institutions 1948–1952, Inclusive. Ann. Surg..

[15] Kawashima Y., Seo N., Morita K., Iwao Y., Irita K., Tsuzaki K., Goto Y., Kobayashi T., Dohi S. (2001). Annual study of perioperative mortality and morbidity for the year of 1999 in Japan: The outlines--report of the Japan Society of Anesthesiologists Committee on Operating Room Safety. Masui Jpn. J. Anesthesiol..

[16] Braz L.G., Braz J.R.C., Modolo M.P., Corrente J.E., Sanchez R., Pacchioni M., Cury J.B., Soares I.B., Braz M.G. (2020). Perioperative and Anesthesia-Related Cardiac Arrest and Mortality Rates in Brazil: A Systematic Review and Proportion Meta-Analysis. PLoS ONE.

[17] Bainbridge D., Martin J., Arango M., Cheng D., Evidence-based Peri-Operative Clinical Outcomes Research (EPiCOR) Group (2012). Perioperative and Anaesthetic-Related Mortality in Developed and Developing Countries: A Systematic Review and Meta-Analysis. Lancet.

[18] Stato—ANIA—Welcome. https://www.ania.it/export/sites/default/it/pubblicazioni/Dossier-e-position-paper/Dossier-ANIA-Malpractice-il-grande-caos.pdf.

[19] Salute M. della Il Programma Nazionale Valutazione Esiti (PNE). http://www.salute.gov.it/portale/temi/p2_6.jsp?id=2905&area=programmazioneSanitariaLea&menu=vuoto.

[20] Salute M. della Normativa. http://www.salute.gov.it/portale/documentazione/p6_2_6.jsp?lingua=italiano&tipo=noCircolari&btnCerca=cerca&iPageNo=216.

[21] Governo Italiano—La Costituzione 2—Titolo V. http://presidenza.governo.it/Governo/Costituzione/2_titolo5.html.

[22] The Economist. 16 May 2015. https://www.economist.com/finance-and-economics/2015/05/16/a-tale-of-two-economies.

[23] Habre W., Disma N., Virag K., Becke K., Hansen T.G., Jöhr M., Leva B., Morton N.S., Vermeulen P.M., Zielinska M. (2017). Incidence of Severe Critical Events in Paediatric Anaesthesia (APRICOT): A Prospective Multicentre Observational Study in 261 Hospitals in Europe. Lancet Respir. Med..

[24] Wolfler A.M., De Silvestri A., Camporesi A., Ivani G., Vittori A., Zadra N., Pasini L., Astuto M., Locatelli B.G., Cortegiani A. (2019). Pediatric Anesthesia Practice in Italy: A Multicenter National Prospective Observational Study Derived from the Apricot Trial. Minerva Anestesiol..

[25] Travis K.W., Mihevc N.T., Orkin F.K., Zeitlin G.L. (1999). Age and Anesthetic Practice: A Regional Perspective. J. Clin. Anesth..

[26] Choudhry N.K., Fletcher R.H., Soumerai S.B. (2005). Systematic Review: The Relationship between Clinical Experience and Quality of Health Care. Ann. Intern. Med..

[27] Tessler M.J., Shrier I., Steele R.J. (2012). Association between Anesthesiologist Age and Litigation. Anesthesiology.

[28] Studdert D.M., Mello M.M., Sage W.M., DesRoches C.M., Peugh J., Zapert K., Brennan T.A. (2005). Defensive Medicine among High-Risk Specialist Physicians in a Volatile Malpractice Environment. JAMA.

[29] Casali M.B., Mobilia F., Sordo S.D., Blandino A., Genovese U. (2014). The Medical Malpractice in Milan-Italy. A Retrospective Survey on 14 Years of Judicial Autopsies. Forensic. Sci. Int..

[30] Genovese U., Blandino A., Midolo R., Casali M.B. (2016). Alleged Malpractice in Anesthesiology: Analysis of a Series of Private Insurance Claims. Minerva Anestesiol..

[31] Hiyama T., Yoshihara M., Tanaka S., Urabe Y., Ikegami Y., Fukuhara T., Chayama K. (2006). Defensive Medicine Practices among Gastroenterologists in Japan. World J. Gastroenterol..

[32] La Medicina Difensiva: Una Ricerca Sul Pronto Soccorso in Italia|Catino|Pratica Medica & Aspetti Legali. https://journals.seedmedicalpublishers.com/index.php/PMeAL/article/view/327/357.

[33] Council of Europe European Commission for the Efficiency of Justice (CEPEJ) https://www.coe.int/en/web/cepej/home.

